# How trust shapes the relationship between employee engagement and organizational citizenship behavior: evidence from Saudi Arabia's private healthcare sector

**DOI:** 10.3389/frhs.2026.1958907

**Published:** 2026-10-29

**Authors:** Abdallah M. Elamin, Ahmed Z. E. Ahmed, Hazem Aldabbas, Liza Gernal

**Affiliations:** College of Business Administration, Fujairah University, Fujairah, United Arab Emirates

**Keywords:** employee engagement, OCB, organizational trust, private healthcare sector, Saudi Arabia, supervisor trust, trust transfer

## Abstract

Employee engagement is known to influence OCB, yet the multilevel trust mechanisms underlying this relationship, particularly in healthcare contexts, remain underexplored. This study examines the direct, parallel, and serial mediating effects of supervisor trust and organizational trust in the link between employee engagement and OCB in Saudi Arabia's private healthcare sector. Grounded in Social Exchange Theory, Leader—Member Exchange Theory, and Trust Transfer Theory, the study tests a conceptual model using PLS-SEM based on a survey of 232 healthcare employees. The findings indicate that employee engagement positively influences OCB both directly and indirectly through supervisor trust and organizational trust. Supervisor trust also has a significant positive effect on organizational trust, reflecting a cascading trust transfer. The model explains 54.5% of the variance in OCB, demonstrating strong explanatory power. The study extends the organizational behavior literature by integrating multilevel trust processes within a single framework that links engagement to discretionary workplace behaviors. The findings offer evidence-based recommendations to strengthen leadership, trust, and employee engagement to improve workforce performance and service excellence within Saudi Arabia's Vision 2030 healthcare transformation agenda.

## 1 Introduction

Employee engagement (EE) is a key concept in human resource management and organizational behavior because it plays a significant role in individual and organizational performance (1). In competitive, service-oriented settings, organizations depend on their staff's technical skills, as well as their physical, mental, and emotional effort. Engagement is a positive, fulfilling state of work that drives people to work with vigor, dedication, and absorption, and to go beyond their formal job requirements (2, 3). Engagement can be a strategic resource for competitive advantage, as highly engaged employees are more committed, resilient, creative, and persistent in their work.

In healthcare, engagement is a key factor because it is associated with the quality of care a healthcare professionals provide, as well as healthcare workers’ attitudes and behaviors (1, 4). Healthcare workers operate in complex, demanding settings with high workloads, emotional demands, interdisciplinary collaboration, and constant patient contact (5). In such situations, employees are expected to perform tasks beyond the scope of their duties (6). These discretionary behaviors are called organizational citizenship behavior (OCB); they are voluntary, help increase organizational effectiveness, and are not formally rewarded (7–9). These include helping others, knowledge sharing, encouraging organizational efforts, and being flexible to patients’ needs (10). Its benefits include better teamwork, service quality, patient satisfaction, and performance (11), which makes its drivers a priority for healthcare organizations aiming for positive patient outcomes (12).

Employee engagement has consistently been identified as a strong predictor of OCB (9). According to Social Exchange Theory (SET), employees are more likely to engage in positive behaviors that benefit the organization when they feel supported and find their work meaningful. As a result, engaged employees are more likely to go the extra mile, collaborate, and give more than expected (3). However, engagement does not necessarily lead to citizenship behavior. Despite high engagement, employees may choose not to contribute if they do not trust their supervisors or the organization (13), suggesting psychological processes that shape this relationship (14).

Trust is a relational mechanism that facilitates cooperation, reduces uncertainty, and enhances knowledge sharing and extra-role behavior (15). Trust can be directed toward multiple organizational objectives, including supervisors and the organization. Trust in supervisors (TS) is shaped by supervisors’ consistent actions demonstrating fairness, competence, integrity, and care for employees’ welfare (16). Organizational trust (OT) is employees’ belief in the organization's fairness and reliability and in its dedication to their interests (17). These two forms affect employee attitudes and behaviors to varying degrees and can interact to influence organizational outcomes (18).

Trust is a critical focus in Saudi Arabia's private healthcare sector, which is undergoing significant transformation under Vision 2030 (19). There is a need to remain ready to adapt to reforms, such as privatization, digitalization, quality improvement, localizing the workforce, and introducing competition, while maintaining high standards of care (20). Other challenges include cultural diversity, communication, leadership, and integration within organizations (4). This dynamic environment fosters supervisor and organizational trust, reducing uncertainty and facilitating engagement in actions that drive colleagues, patients, and service excellence (21).

Although substantial research has examined EE, trust, and OCB, several gaps remain. First, prior work has primarily focused on direct links between engagement and OCB, leaving the underlying mechanisms underexplored (3, 22). Second, studies have examined supervisor and organizational trust separately rather than together (23). Third, a key gap concerns how employee engagement translates into broader OCB through multiple trust mechanisms. Although prior studies have examined individual trust pathways (24, 25), parallel and serial mechanisms operating simultaneously remain underexplored. For instance, Aldabbas et al. (17) examined only managerial trust, organizational trust, and sportsmanship. Fourth, empirical evidence in Saudi Arabia, particularly in the private healthcare sector, is limited, raising concerns about generalizing findings from other contexts (26).

Accordingly, this study aims to fill these gaps by examining how employee engagement influences OCB directly and through multilevel trust mechanisms in Saudi Arabia's private healthcare sector. Specifically, it examines the relationships among employee engagement, trust in the supervisor, organizational trust, and OCB; evaluates the parallel mediating roles of supervisor and organizational trust; and investigates the sequential trust-transfer mechanism through which trust in the supervisor develops into organizational trust and subsequently promotes OCB. Drawing on SET, Leader–Member Exchange (LMX) theory, and Trust Transfer Theory, a multilevel construct is developed, including reciprocity, high-quality relations between supervisors and employees, and cross-level trust, to explain how engagement leads to discretionary behavior (27, 28). This study builds on earlier work that examined each mechanism individually (29, 30). It provides evidence in the context of Vision 2030 healthcare reform of the impact of multilevel trust and engagement on OCB and service excellence.

## 2 Saudi Arabia's healthcare transformation

Saudi Arabia's Vision 2030 and the Health Sector Transformation Program advocate transforming both the healthcare delivery system and leadership through privatization, digitalization, and localization of human resources (19, 31). These changes must continuously adapt to evolving systems and performance expectations (32). Trust is a key asset for an organization in a dynamic environment. Supervisors and institutions can provide clarity and support during periods of change, and discretionary contributions stem from engagement, allowing actions beyond the formal job role (3). Building trust-based relationships is thus critical to maintaining collaboration and organizational citizenship behavior in service-rich environments (31). The sector's cultural diversity, contractual employment, and localization approaches shape trust development (4, 19). While professionals have high levels of intrinsic engagement, this may not yet translate into discretionary action, which is grounded in trust (4). Analyzing the connection between trust, engagement, and organizational citizenship behavior remains important for addressing readiness and coordination issues (19).

## 3 Theoretical framework

The study has employed Social Exchange Theory (SET), Leader–Member Exchange (LMX) theory, and Trust Transfer Theory to explain how employee engagement can lead to organizational citizenship behavior (OCB) through multilevel trust mechanisms (27, 28).

The primary theoretical model is SET, which posits that workplace relationships are dynamic exchanges in which workers respond to positive treatment with positive attitudes and discretionary behavior (16, 33, 34). Recent literature indicates that trust and reciprocity positively influence employees’ satisfaction and commitment when organizations and supervisors provide support (35). In healthcare, where collaboration and volunteer effort are essential to providing patient care, employees are more likely to cooperate, engage, and demonstrate citizenship behavior if they feel supported by the organization, treated fairly in processes, and trusted by its management (36, 27);. Therefore, SET clarifies the mechanism by which supportive organizational and supervisory relationships can drive employee engagement and increase employees’ trust in supervisors and the organization, leading to OCB (29).

LMX theory can be used alongside SET to understand how the quality of supervisor–employee relationships influences work attitudes and behaviors (37). Unlike SET, which emphasizes reciprocal exchanges, LMX focuses on dyadic leader–follower relationships grounded in mutual trust, respect, and reciprocal obligation (34). Meta-analytic research shows that high-quality LMX relationships are positively associated with employee performance, satisfaction, organizational commitment, and role clarity, and negatively associated with intentions to leave (38) (39);. These ties tighten in inclusive organizational cultures and loosen when exchanges are low quality (40). In healthcare, supervisory relationships are a key factor because trust is directly linked to patient safety and service quality (41, 42). Trust in supervisors is related to organizational citizenship behavior and mediates the relationship between leadership and extra-role behaviors (13). LMX is linked to citizenship behavior, which in turn relates to employee engagement (43). Thus, LMX clarifies how supportive supervision fosters trust and discretionary employee behavior.

Building on these ideas, Trust Transfer Theory proposes that trust can be transferred from one organizational actor to another, such as from a supervisor to the organization (24, 28). Supervisors are the face of the organization in day-to-day relationships with employees, so perceptions of supervisors’ fairness, integrity, and support can influence general trust in the organization (17). This mechanism is especially relevant in health-care settings, where supervisors communicate policies, facilitate collaboration among workers, and help them navigate uncertainty (31). Therefore, positive supervisor relationships can build interpersonal trust, which can further enhance organizational trust and, consequently, result in discretionary behaviors such as OCB (44). Studies also show that trust can be passed to other organizational units and the institutional level through chain trust (45–47). Using Trust Transfer Theory alongside SET and LMX provides a holistic, multi-level understanding of how employee engagement boosts supervisor trust, which in turn increases trust in the organization and, as a result, improves OCB.

## 4 Literature review and hypotheses development

### 4.1 Employee engagement in healthcare

Employee engagement is a positive, fulfilling psychological state related to work, characterized by vigor, dedication, and absorption, and it reflects employees’ cognitive, emotional, and physical involvement in work (2, 48, 49). Employee engagement is distinct from job satisfaction or organizational commitment; it involves active participation, enthusiasm, and a commitment to putting in effort beyond what is expected to accomplish organizational goals (50) (51);. In healthcare, engagement is more than satisfaction with pay or job security; it involves healthcare professionals’ dedication to high-quality patient care and supporting organizational goals. Evidence shows that employee engagement is linked to supportive leadership, participative decision-making, opportunities for professional development, and organizational support, which, in turn, lead to service quality, patient safety, and overall organizational performance (1, 4). Therefore, employee engagement is a valuable organizational resource that can help maintain a quality health care system and enhance patient health.

### 4.2 Organizational citizenship behavior in healthcare

Organizational citizenship behavior (OCB) refers to behaviors employees perform that are not part of their formal job duties or explicitly rewarded but help the organization function (7, 8). OCB goes beyond the job scope and includes assisting coworkers, mentoring new members, transferring knowledge, participating in quality improvement projects, and maintaining professionalism in challenging situations (11). In the healthcare sector, these behaviors are particularly relevant because they can improve collaboration among healthcare providers, teamwork, and care coordination, ultimately enhancing patient safety, service quality, and organizational performance (9). Furthermore, it is crucial to differentiate between organizational citizenship behavior toward the organization (OCB-O) and organizational citizenship behavior toward individuals (OCB-I), because each may be affected by different organizational and relational factors (18). Both types of OCB are essential to providing effective healthcare services.

### 4.3 Trust in supervisor and organizational trust in healthcare

Trust Transfer Theory suggests that trust established in one relationship can transfer to a related target, such as from the supervisor to the organization (24, 28, 52). Trust in the supervisor is linked to perceived competence, integrity, and benevolence, whereas organizational trust is linked to perceived fairness, reliability, and care for employees’ interests (42, 47, 53, 54). Employees draw on their experiences with their supervisor to inform their experiences with the organization (28, 52). This knowledge transfer can be highly valuable in the healthcare industry, where supervisors can share policies, provide assistance, and reduce anxiety in difficult clinical situations (21). Psychological safety and felt obligation are the two channels through which trust in supervisors positively affects engagement and organizational commitment, which, in turn, promotes organizational trust and, through social exchange processes such as perceived organizational support, leads to the development of OCB (13, 24, 36, 41). Thus, the proposed sequence of supervisor trust, organizational trust, and OCB is theorized on the basis of the Trust Transfer Theory.

### 4.4 Employee engagement and organizational citizenship behavior

Employee engagement is a key factor influencing organizational citizenship behavior (OCB), particularly in healthcare, because employees’ discretionary actions affect teamwork, service quality, and patient outcomes (9). Engaged employees are more willing to invest cognitive, emotional, and physical effort beyond their job duties, which manifests in voluntary actions that support organizational effectiveness (3). Meta-analytic evidence also suggests that the engagement–OCB relationship is mediated by broader attitudinal mechanisms, such as organizational commitment and job satisfaction (22). In healthcare, citizenship behaviors are important for successful clinical coordination and patient safety, including willingness to help colleagues, working as a team under pressure, and participating in organizational improvement (55). Empirical studies have found that high levels of healthcare employee engagement are linked to increased organizational and interpersonal citizenship behaviors (56). Therefore, the following hypothesis is proposed:

H1: Employee engagement has a positive effect on organizational citizenship behavior.

### 4.5 Employee engagement and trust dimensions

Employee engagement will enhance trust in supervisors and the organization in distinct yet complementary ways. In the Leader–Member Exchange (LMX) theory, an engaged employee demonstrates commitment, responsiveness, and cooperation with their supervisor, building a high-quality exchange relationship characterized by trust and respect (34). Organizational-level engagement shapes employees’ perceptions of organizational actions and managerial decisions. High engagement is linked to more positive attitudes toward policies, leadership intentions, and workplace practices, which boost organizational trust (47, 57). Leadership studies also support these relationships, highlighting the positive effects of leadership humility, transformational leadership, and coaching leadership on employee engagement and work attitudes (16, 58). Engagement is particularly relevant in healthcare, where it is essential to build trust with supervisors and the organization amid challenging work. Employees’ engagement levels positively influence their perceptions of leadership and organizational practices, ultimately leading to greater trust among employees and improved collaboration and service quality (59, 60). Therefore, the following hypotheses are proposed:

H2: Employee engagement has a positive effect on trust in supervisor.
H3: Employee engagement has a positive effect on organizational trust.

### 4.6 Trust dimensions and organizational citizenship behavior

Supervisors’ trust and organizational trust lead to organizational citizenship behavior (OCB) in complementary ways. Supervisors’ trust most strongly affects interpersonal citizenship behaviors (helping and teamwork). Perceiving supervisors as fair, competent, and supportive increases employees’ psychological safety, which in turn increases the likelihood of discretionary helping behavior and cooperation beyond the formal workplace (61). Higher levels of supervisor trust improve collaboration, information sharing, and extra-role performance at work (62, 63). Organizational trust, however, supports organization-directed citizenship behaviors such as conscientiousness, civic involvement, and sportsmanship. Employees feel loyal and committed to an organization that treats them fairly and recognizes their contributions, especially during uncertainty (29). This distinction matters in healthcare, where providing health services depends on collaboration and discretionary actions to achieve goals and ensure continuity of care (44, 64). Evidence of these patterns has been observed. Monitoring styles are associated with trust, which in turn positively affects OCB and innovation (65). Supervisory justice and perceived supervisor support, which enhance OCB, are associated with supervisor trust (66). At the organizational level, justice, workplace spirituality, and organizational trust are positively related to commitment and citizenship behaviors (18). In healthcare settings, organizational trust is paramount, safeguarding well-being and maintaining behaviors when faced with stressors (15). Therefore, the following hypotheses are proposed:

H4: Trust in supervisor has a positive effect on organizational citizenship behavior.
H5: Organizational trust has a positive effect on organizational citizenship behavior.

### 4.7 Trust in supervisor as antecedent to organizational trust

Trust in supervisors is an important antecedent of organizational trust because employees often generalize supervisory experiences to the organization they represent (47). As the face of the organization, supervisors shape employees’ assessments of organizational trustworthiness through their fairness, integrity, transparency, and support (17, 47). This is particularly relevant in hierarchical settings, where supervisors distribute resources, communicate expectations, and clarify policies, thereby shaping employees’ workplace experiences (67). Empirical evidence indicates that as perceived supervisor support increases, leader-member exchange and supervisor trust rise, which in turn fosters a positive relationship with employee attitudes (23). Trust-transfer research also shows that trust can transfer from a relational to an organizational level (46). Trust in the supervisor–employee relationship therefore positively influences overall organizational trust, which in turn leads to better organizational outcomes, such as organizational citizenship behavior (17, 68). Therefore, the following hypothesis is proposed:

H6: Trust in supervisor has a positive effect on organizational trust.

### 4.8 Parallel and serial mediation: trust in supervisor and organizational trust

Building on the constituent relationships proposed in H1–H6, the following hypotheses focus on the mechanisms through which employee engagement translates into OCB. More specifically, this study assumes that employee engagement affects OCB through parallel and serial trust-based mechanisms grounded in Social Exchange Theory (SET), LMX theory, and Trust Transfer Theory. In the parallel model, the study conceptualizes direct relationships between engagement and supervisor and organizational trust, and between each and OCB. The serial model, by contrast, posits that engagement increases trust in supervisors, which translates into increased trust in the organization and ultimately into OCB. Both pathways can be examined to understand how positive employee experiences relate to discretionary actions (30, 69).

As in SET and LMX, supportive work environments foster positive employee attitudes and discretionary behaviors, and several mechanisms can operate simultaneously. For example, organizational trust, justice, and identification each mediate the relationship between micro-corporate social responsibility and employee outcomes (70). Similarly, organizational justice is a parallel mediator between diversity management and OCB (30). In organizations, justice and work engagement are two parallel mechanisms that simultaneously mediate the pathway between inclusive leadership and OCB in the health care sector (71). Based on H2, H4, H3, and H5, this study argues that trust in the supervisor and trust in the organization are complementary paths that will lead to an increased level of OCB; trust in the supervisor will increase cooperation, helping, and knowledge sharing, and trust in the organization will increase identification, commitment, and organization-directed citizenship (29, 34, 72). Accordingly:

H7: Trust in supervisor mediates the relationship between employee engagement and organizational citizenship behavior.
H8: Organizational trust mediates the relationship between employee engagement and organizational citizenship behavior.

Beyond their independent mediating roles, supervisor and organizational trust may operate sequentially. Trust Transfer Theory elaborates this serial pathway. As representatives of the organization, supervisors’ positive experiences are generalized to the organization's overall experience (17, 28). Trust in supervisors is therefore further reinforced, creating a ripple effect that fosters trust within the organization and ultimately results in OCB. Empirical evidence supports this mechanism. Trust mediates the relationships between leadership and its outcomes, such as psychological safety and empowerment (24); knowledge-oriented leadership and innovative performance (25); and affective supervisor trust and OCB (73). In healthcare, engagement is associated with OCB (74). These studies show that interpersonal and organizational processes are interrelated and occur during trust development (75). Together, SET, LMX, and Trust Transfer Theory provide a solid theoretical basis for studying independent and sequential trust mechanisms and for understanding the process of engagement to OCB (29, 76). Accordingly:

H9: Trust in supervisor and organizational trust sequentially mediate the relationship between employee engagement and organizational citizenship behavior.

### 4.9 Integrated theoretical framework and conceptual model

The proposed framework integrates employee engagement (EE), trust in supervisor (TS), organizational trust (OT), and organizational citizenship behavior (OCB) within a relational model grounded in social exchange theory, leader–member exchange theory, and trust transfer theory. Figure 1 shows that EE is both a direct and an indirect (trust-mediated) antecedent of OCB. Specifically, EE is expected to positively affect OCB (H1) and to strengthen TS (H2) and OT (H3). It is hypothesized that TS will directly influence OCB (H4) and positively influence OT (H6), while OT positively influences OCB (H5). The model also includes two mediation structures: two complementary mediation relationships between EE and OCB through TS and OT (H7 and H8), and one serial mediation relationship between EE and OCB through TS and OT (H9). This framework offers a combined explanation of how engagement can lead to discretionary employee behaviors via interrelated interpersonal and organizational trust processes in Saudi private healthcare. Collectively, H1–H9 specify an integrated framework in which employee engagement influences OCB directly and through parallel and sequential trust mechanisms, as illustrated in Figure 1.

**Figure 1:**
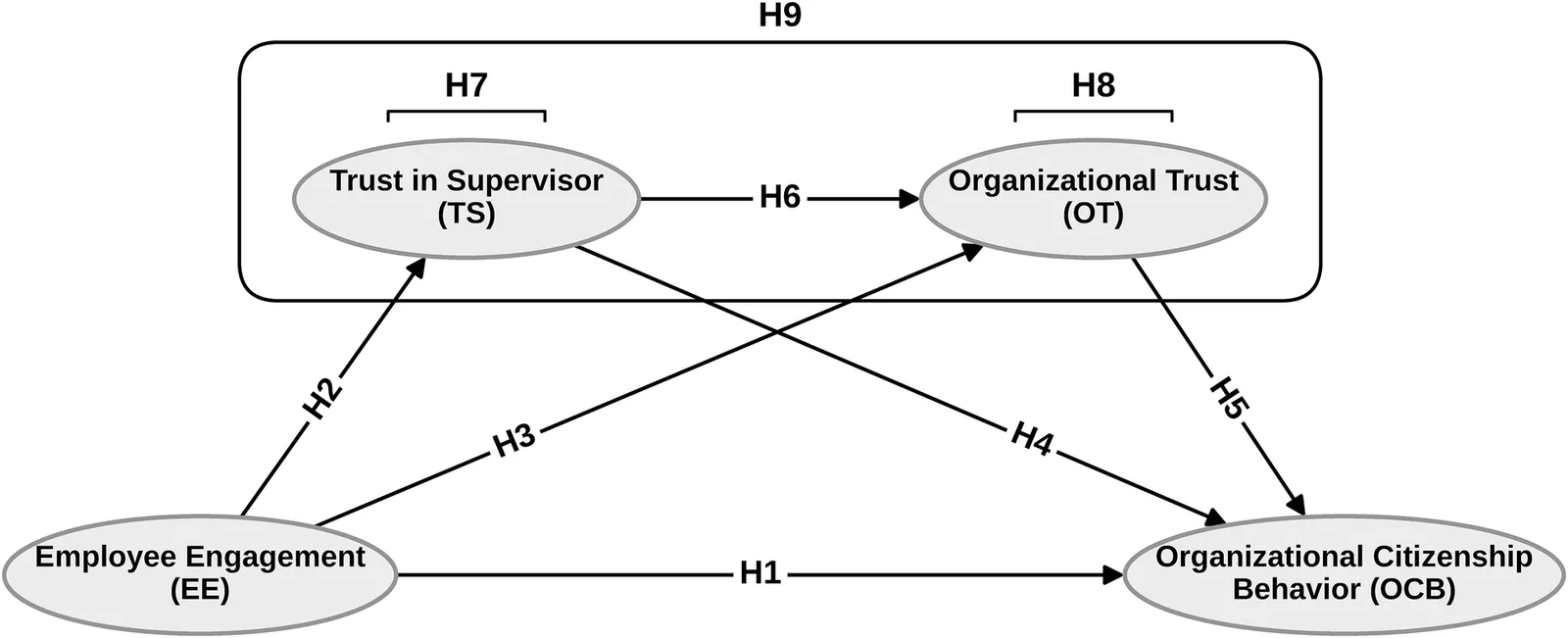
Conceptual model. EE, Employee Engagement; TS, Trust in Supervisor; OT, Organizational Trust; OCB, Organizational Citizenship Behavior. The model specifies direct relationships and parallel mediation through TS and OT, along with the serial trust-transfer pathway EE→TS→OT→OCB.

## 5 Methodology

### 5.1 Research design and data collection

This study adopted a quantitative approach, using a cross-sectional survey design to examine the relationships among employee engagement, trust in the supervisor, organizational trust, and organizational citizenship behavior (OCB) in the Saudi Arabian private healthcare sector. A quantitative approach can test theoretical relationships among latent constructs and estimate the strength of their associations (77). The cross-sectional design enabled efficient data collection within the dynamic organizational context and provided insights into employees’ perceptions. Convenience sampling was used, a common method in organizational studies when access to respondents is limited (78, 79).

Of the 500 questionnaires distributed, 251 were returned. After excluding 19 responses for excessive brevity, incompleteness, or straight-lining, 232 valid responses were retained, yielding a response rate of 46.4%. To ensure transparency regarding sample independence, the dataset used in the present study was collected specifically for this research and is entirely distinct from the dataset analyzed by Aldabbas et al. (17). Whereas the earlier study examined 287 employees from multiple industries in Riyadh and the Western Province, the present study comprises 232 employees exclusively from private healthcare organizations in Riyadh and the Eastern Province. No respondents or analytical data are shared between the two studies. Participation was voluntary, anonymous, and confidential, and respondents were informed of their right to withdraw at any time. An *a priori* power analysis conducted using G*Power 3.1 (f^2^ = 0.08, *α* = 0.05, statistical powe*r* = 0.95) indicated a minimum required sample size of 219 (80). The final sample of 232 therefore exceeded this requirement, providing adequate statistical power for hypothesis testing. The study was reviewed and approved by the Research Ethics Committee of Fujairah University, United Arab Emirates (Approval No. UOF/REC/2025-02-15), in accordance with the institutional requirements of the authors’ host institution; this approval applies to the United Arab Emirates only. All data were collected in Saudi Arabia, where ethics approval was not required, since this is a questionnaire-based study with no invasive procedures that does not deal with a sensitive topic. No patient data or identifying information were collected, and written informed consent was obtained from all participants.

### 5.2 Sample composition and demographics

Table 1 presents the demographic and occupational profile of the 232 respondents. The sample was nearly balanced by gender, with 50.9% male and 49.1% female. More than half of respondents (59.1%) were 35 years or younger, and 58.6% were non-Saudi employees, indicating that Saudi Arabia's private healthcare sector is multicultural. Furthermore, 61.2% held a first degree or higher, and 47.8% had five years or less in the organization. Most participants were non-managerial (65.1%), and the largest group was clinical staff (59.1%). In terms of workplace setting, 55.2% practiced in hospitals, 25.0% in medical centers, and 19.8% in clinics. Overall, the sample is fairly representative of the private healthcare sector and provides a suitable foundation for exploring employee engagement, trust, and organizational citizenship behavior in this setting.

**Table 1:** Demographic and occupational characteristics of the sample.

	Frequency	
Variables	n (total = 232)	% (total = 100%)
Gender		
Male	118	50.9
Female	114	49.1
Age		
35 years or less	137	59.1
36–46	75	32.3
47 years and above	20	8.6
Nationality		
Saudi	96	41.4
Non-Saudi	136	58.6
Educational Level		
High School or less	31	13.4
Diploma	59	25.4
First Degree and above	142	61.2
Organizational Tenure		
5 years and less	111	47.8
6–10 years	75	32.3
11 years and more	46	19.8
Managerial Level		
Non-Managerial	151	65.1
Supervisory Management	41	17.7
Middle Level Management	28	12.1
Top Management	12	5.2
Job Category		
Clinical staff	137	59.1
Non-Clinical Staff	95	40.9
Healthcare Facility		
Hospital	128	55.2
Medical Center	58	25.0
Clinic	46	19.8

### 5.3 Measurement

Construct validity and reliability were established using established, validated multi-item scales for all constructs. Employee Engagement (EE), Trust in Supervisor (TS), Organizational Trust (OT), and Organizational Citizenship Behavior (OCB) were measured with scales adapted from prior studies. To enhance contextual relevance while preserving conceptual equivalence in the Saudi Arabian context, we made minor wording adjustments (81). All items were rated on a 5-point Likert scale (1 = strongly disagree, 2 = disagree, 3 = neither agree nor disagree, 4 = agree, 5 = strongly agree).

The Utrecht Work Engagement Scale (UWES-9), developed by Schaufeli et al. (82), was used to assess employee engagement. The scale measures the three dimensions of engagement, namely vigor, dedication, and absorption, and a higher-order engagement construct was modeled (83). Examples of these items are: “At my work, I feel bursting with energy”; “I am enthusiastic about my job”; and “I feel happy when I am working intensely.” Organizational citizenship behavior was assessed using the instrument developed by Niehoff and Moorman (84), which was later adapted by Tziner and Sharoni (85) and Elamin and Tlaiss (86). The scale rates five dimensions, namely conscientiousness, sportsmanship, civic virtue, courtesy, and altruism. Examples of these items are: “I have not taken an additional break”; “I have been complaining about unimportant things” (reverse coded); “I have been attending meetings that, although not required, were important”; “I did not violate others’ rights”; and “I helped others who had heavy workloads.” In line with Kabasakal et al. (87), employee self-reported OCB was evaluated and treated as a higher-order construct. Multisource assessments are recommended, but self-report measures are suitable because OCB involves discretionary behaviors directed at more than one recipient and across multiple contexts, which are not likely to be comprehensively evaluated by any one source (88, 89). Following the work of Gupta and Singh (90), OCB was measured as employees’ self-reported discretionary behaviors that aid the organization. Trust in Supervisor was assessed using the six-item scale designed by Podsakoff et al. (91), which measures employees’ trust in their supervisors’ fairness, integrity, loyalty, honesty, and reliability. Trust in supervisors is defined as employees’ expectation that supervisors will be fair to them and treat them well (92). A representative item is “I am pretty certain that my boss always tries to treat me fairly.” Organizational Trust was measured using a seven-item scale assessing employees’ confidence in the organization's integrity, reliability, and commitment to fulfilling its duties (53, 93). A representative item is “I think that my employer is honest.”

To ensure linguistic and cultural appropriateness, independent bilingual experts translated the questionnaire into Arabic and back-translated it, resolving discrepancies to achieve semantic equivalence (94). Content validity was established by three academic experts and two industry practitioners, who evaluated item relevance, clarity, and contextual appropriateness. The researchers also pilot-tested readability and comprehensibility with 15 participants. The instrument was revised based on feedback from both phases to further enhance its clarity, contextual fit, and cultural appropriateness for the Saudi Arabian healthcare setting.

### 5.4 Analytical approach

Hair et al. (95) indicate that PLS-SEM is appropriate for complex models with multiple mediators and predictive pathways and can be used for explanation and prediction, so it was deemed appropriate for the data analysis. It is also applicable to data that do not necessarily have a normal distribution and to research in new and/or situation-specific contexts (96). Reliability of the measurement model (Cronbach's alpha, composite reliability) and convergent and discriminant validity were tested. Path coefficients, effect sizes, R^2^, adjusted R^2^, and predictive relevance (Q^2^_predict) were used to evaluate the structural model. This approach allows extensive evaluation of the model's validity, explanatory power, and predictive accuracy.

## 6 Results

### 6.1 Measurement model assessment

The measurement model was evaluated using indicator loadings, Cronbach's alpha, composite reliability (rho_C), rho_A, and average variance extracted (AVE) Table 2). Most indicators exceeded the recommended loading threshold of 0.708, supporting adequate indicator reliability. However, one OCB item (OCBS) had a low loading (0.578) and was removed after further assessment of its reliability. Re-estimating the model after its removal improved the psychometric properties of the OCB construct: Cronbach's alpha increased from 0.853 to 0.888, rho_A from 0.863 to 0.889, rho_C from 0.897 to 0.923, and AVE from 0.641 to 0.749. In the revised measurement model, Cronbach's alpha ranged from 0.888 to 0.943, rho_A from 0.889 to 0.949, and rho_C from 0.920 to 0.952, all exceeding the recommended threshold of 0.70. Similarly, AVE values ranged from 0.622 to 0.749, surpassing the recommended minimum of 0.50 and supporting convergent validity (95). Collectively, these results demonstrate satisfactory indicator reliability, internal consistency, and convergent validity across all constructs.

**Table 2:** Reliability and convergent validity.

Construct	Item	Loading	Cronbach's Alpha	Composite Reliability	(rho_a)	AVE
EE	EE1	0.771	0.943	0.952	0.949	0.689
	EE2	0.844				
	EE3	0.836				
	EE4	0.861				
	EE5	0.823				
	EE6	0.764				
	EE7	0.829				
	EE8	0.844				
	EE9	0.889				
TS	TS1	0.874	0.922	0.939	0.924	0.720
	TS2	0.876				
	TS3	0.853				
	TS4	0.841				
	TS5	0.847				
	TS6	0.796				
OT	OT1	0.739	0.898	0.920	0.901	0.622
	OT2	0.847				
	OT3	0.785				
	OT4	0.752				
	OT5	0.804				
	OT6	0.829				
	OT7	0.759				
OCB	OCBA	0.864	0.888	0.923	0.889	0.749
	OCBC	0.864				
	OCBCR	0.883				
	OCBCV	0.850				

Discriminant validity was assessed using the heterotrait–monotrait ratio (HTMT) and the Fornell–Larcker criterion. HTMT values for all constructs were below the conservative threshold of 0.85, indicating satisfactory construct distinctiveness, as shown in Table 3. Furthermore, the square root of AVE for each construct exceeded the correlations between that construct and the other constructs, as shown in Table 4 (Fornell-Larcker criterion), indicating that each construct explained more variance through its own indicators than through other constructs. Overall, these results indicate good psychometric properties and provide evidence of discriminant validity in accordance with Hair, Black, Baburam, and Cortham (95).

**Table 3:** HTMT values.

Construct	EE	TS	OT	OCB
EE				
TS	0.432			
OT	0.445	0.690		
OCB	0.576	0.693	0.702	

**Table 4:** Fornell-larcker criterion.

Construct	EE	TS	OT	OCB
EE	0.830			
TS	0.411	0.848		
OT	0.417	0.636	0.789	
OCB	0.535	0.631	0.630	0.865

#### 6.1.1 Assessment of common method bias (CMB)

Common method bias (CMB) was carefully assessed both procedurally and statistically given the cross-sectional, self-reported nature of the data. Assurances of anonymity and confidentiality were provided to reduce the risk of social desirability bias and respondents’ evaluation apprehension (97). Harman's single-factor test showed that the first factor explained 37.5% of the total variance, which did not exceed 50%, so there was no significant concern regarding CMB (98).

### 6.2 Model fit assessment

The standardized root mean square residual (SRMR) and the normed fit index (NFI) were used to assess overall model fit. The model showed acceptable fit, with an SRMR of 0.060, below the recommended value of 0.08 (99), indicating that the model accounted for the observed and model-implied correlation matrix satisfactorily. The NFI value of 0.824 is below the desired threshold but remains acceptable as a supplementary fit index in complex PLS-SEM models (100). Overall, these indices indicate that the proposed model has acceptable global fit and is suitable for further structural model assessment.

### 6.3 Structural model assessment

#### 6.3.1 Collinearity assessment

Collinearity was evaluated prior to hypothesis testing using variance inflation factors (VIFs) (Table 5). The outer VIFs ranged from 1.989 to 4.942, all below the recommended cutoff of 5.0, indicating no problematic multicollinearity among indicators (95). Inner VIFs ranged from 1.000 to 1.764, well below the conservative value of 3.3 recommended for rigorous research (95). In addition, the low VIFs for exogenous constructs indicate that common method bias is not a serious threat to the validity of the results.

**Table 5:** Collinearity statistics for measurement and structural models.

Construct/Path	Indicator/Predictor	VIF
Outer Model (Measurement)		
EE	EE1—EE9	2.495–4.942
TS	TS1—TS6	1.989–3.136
OT	OT1—OT7	2.178–2.867
OCB	OCBA, OCBC, OCBCR, OCBCV	2.153–2.711
Inner Model (Structural)		
EE → OCB	EE	1.265
EE→ TS	EE	1.000
EE→ OT	EE	1.203
TS→ OT	TS	1.203
TS → OCB	TS	1.754
OT → OCB	OT	1.764

#### 6.3.2 Structural model quality (endogenous constructs)

The structural model's explanatory and predictive power was assessed using R^2^, adjusted R^2^, and Q^2^_predict. As shown in Table 6, employee engagement explains 16.9% of the variance in trust in supervisor (R^2^ = 0.169; R^2^_adj = 0.165), indicating modest explanatory power. It also explains 43.3% of the variance in organizational trust (R^2^ = 0.433; R^2^_adj = 0.428) and 54.5% of the variance in organizational citizenship behavior (R^2^ = 0.545; R^2^_adj = 0.539). In addition, all Q^2^_predict values are above zero for trust in supervisor (Q^2^ = 0.155), organizational trust (Q^2^ = 0.159), and organizational citizenship behavior (Q^2^ = 0.272), confirming predictive relevance. Overall, these findings indicate that the proposed model is satisfactory for explaining and predicting focal relationships in the private healthcare sector in Saudi Arabia.

**Table 6:** Structural model quality (endogenous constructs).

Endogenous Constructs	$R^{2}$	$R_{Adj}^{2}$	$Q_{Predict}^{2}$
TS	0.169	0.165	0.155
OT	0.433	0.428	0.159
OCB	0.545	0.539	0.272

#### 6.3.3 Mediation Effects and Hypothesis Testing

Bootstrapping in SmartPLS with 5,000 resamples was used to assess structural relationships and mediation effects. Figure 2 displays the results, and Table 7 presents the hypothesis test results. All hypothesized paths were statistically significant, with 95% bias-corrected confidence intervals that did not include zero, providing strong support for the proposed model.

**Figure 2:**
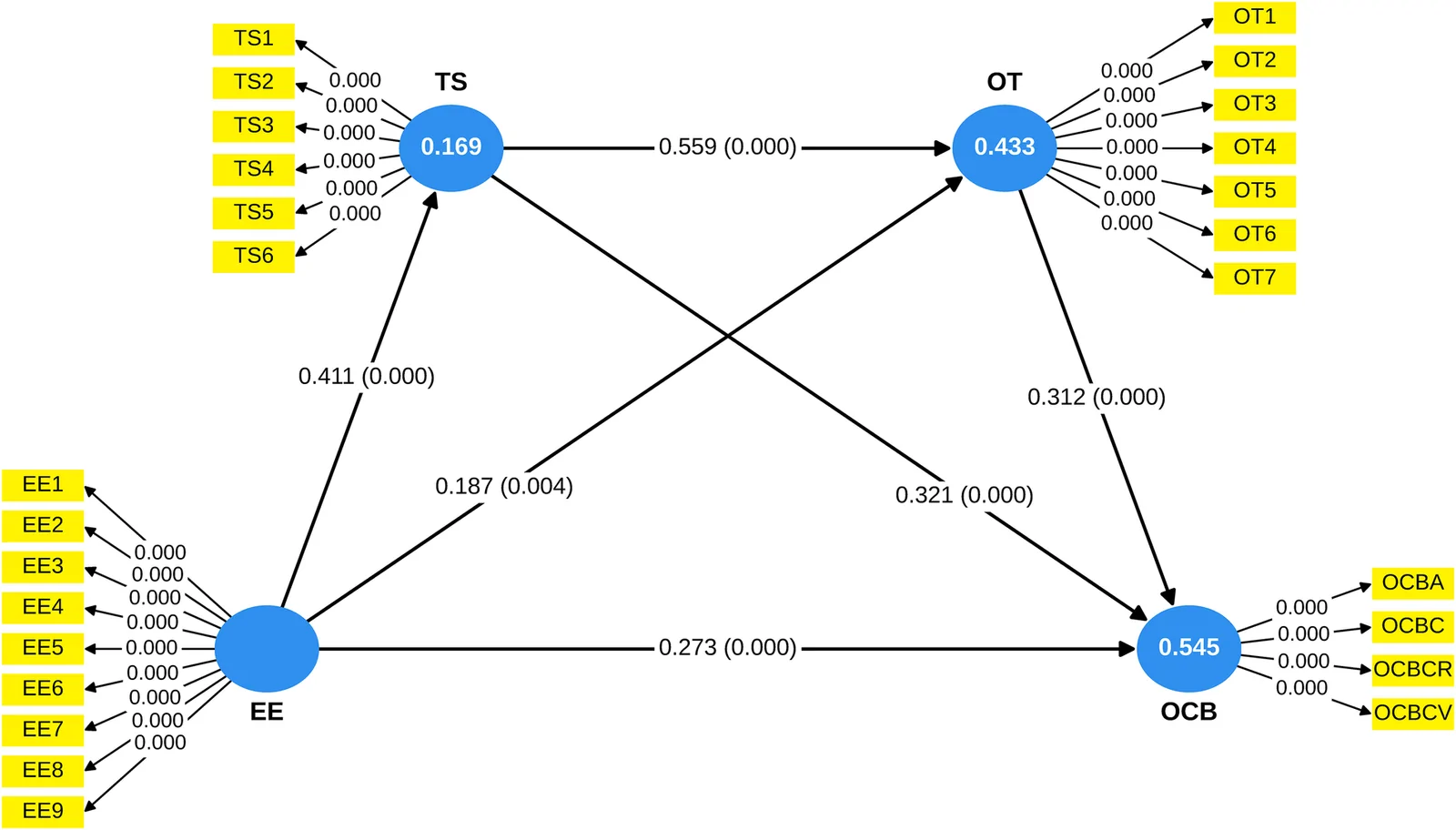
Results of the structural model assessment.

**Table 7:** Mediation effects & hypothesis testing.

Relationship	*Path (β)*	*t-value*	*p-value*	*95% CI*	$f^{2}$	Result
Direct Effects						
H1: EE→OCB	0.273	5.061	0.000	[0.167, 0.376]	0.130	Supported
H2: EE→TS	0.411	6.037	0.000	[0.260, 0.533]	0.203	Supported
H3: EE→OT	0.187	2.876	0.004	[0.058, 0.313]	0.051	Supported
H4: TS→OCB	0.321	3.923	0.000	[0.156, 0.480]	0.129	Supported
H5: OT→OCB	0.312	4.318	0.000	[0.170, 0.451]	0.122	Supported
H6: TS→OT	0.559	9.557	0.000	[0.432, 0.662]	0.457	Supported
Indirect Effects						
H7: EE→TS→OCB	0.132	3.407	0.001	[0.063, 0.216]		Supported/ Partial Mediation
H8: EE→OT→OCB	0.059	2.753	0.006	[0.024, 0.112]		Supported/ Partial Mediation
H9: EE→TS→OT→OCB	0.072	2.898	0.004	[0.032, 0.128]		Supported/ Partial Mediation

Regarding the direct effects, employee engagement (EE) had a significant positive effect on organizational citizenship behavior (OCB) (*β* = 0.273, t = 5.061, *p* < 0.001, 95% CI [0.167, 0.376], f^2^ = 0.130), supporting H1. EE also positively influenced trust in supervisor (TS) (*β* = 0.411, t = 6.037, *p* < 0.001, 95% CI [0.260, 0.533], f^2^ = 0.203), supporting H2, and organizational trust (OT) (*β* = 0.187, t = 2.876, *p* = 0.004, 95% CI [0.058, 0.313], f^2^ = 0.051), supporting H3.

TS had a significant positive effect on OCB (*β* = 0.321, t = 3.923, *p* < 0.001, 95% CI [0.156, 0.480], f^2^ = 0.129), supporting H4, and a strong positive effect on OT (*β* = 0.559, t = 9.557, *p* < 0.001, 95% CI [0.432, 0.662], f^2^ = 0.457), supporting H6. The TS→OT relationship was the strongest structural path and had the largest effect size in the model, highlighting the important role of supervisory trust in fostering broader organizational trust. OT also significantly enhanced OCB (*β* = 0.312, t = 4.318, *p* < 0.001, 95% CI [0.170, 0.451], f^2^ = 0.122), supporting H5. Collectively, these findings demonstrate that both supervisor-level and organization-level trust contribute significantly to employees’ citizenship behavior.

The mediation analysis further revealed significant indirect effects of EE on OCB through both trust mechanisms. Specifically, the indirect effect through TS (EE→TS→OCB) was positive and significant (*β* = 0.132, t = 3.407, *p* = 0.001, 95% CI [0.063, 0.216]), supporting H7. Similarly, the indirect effect through OT (EE→OT→OCB) was significant (*β* = 0.059, t = 2.753, *p* = 0.006, 95% CI [0.024, 0.112]), supporting H8. Furthermore, the serial mediation pathway EE→TS→OT→OCB was significant (*β* = 0.072, t = 2.898, *p* = 0.004, 95% CI [0.032, 0.128]), supporting H9. Thus, all three indirect pathways were statistically significant, as their 95% bootstrap confidence intervals excluded zero.

Importantly, the direct EE→OCB relationship remained significant after accounting for the mediating mechanisms (*β* = 0.273, *p* < 0.001), indicating complementary partial mediation. These findings suggest that employee engagement promotes OCB not only directly but also through parallel and sequential trust mechanisms. Specifically, engagement strengthens supervisor trust and organizational trust independently, while also initiating a trust-transfer process in which greater supervisor trust fosters organizational trust, which subsequently promotes citizenship behavior.

#### 6.3.4 Measurement invariance and multigroup analysis

Given the sample's culturally diverse composition, we conducted additional analyses to assess whether the proposed relationships differed between Saudi (41.4%) and non-Saudi (58.6%) employees. Before conducting multigroup analysis (MGA), we applied the Measurement Invariance of Composite Models (MICOM) procedure. We established configural invariance by using identical indicators, model specifications, and estimation procedures across both groups.

As shown in Table 8 –Panel A, the MICOM analysis established compositional invariance for OCB (c = 1.000, *p* = 0.492), OT (c = 1.000, *p* = 0.697), and TS (c = 1.000, *p* = 0.685). Equality of composite means and variances was supported for OCB and TS, indicating full measurement invariance. For OT, equality of variances was supported (*p* = 0.335), whereas composite means differed significantly (*p* = 0.021), supporting partial measurement invariance. Conversely, we did not establish compositional invariance for EE (c = 0.994, *p* = 0.010), despite nonsignificant differences in its composite mean (*p* = 0.386) and variance (*p* = 0.398). Accordingly, cross-group comparisons involving EE were interpreted cautiously.

**Table 8:** Measurement invariance and multigroup analysis across Saudi and non-Saudi employees.

Panel A. MICOM Assessment.					
Construct	Original Correlation	Compositional Invariance p	Mean Difference p	Variance Difference p	Invariance Conclusion
EE	0.994	0.010	0.386	0.398	Not established
OCB	1.000	0.492	0.434	0.115	Full
OT	1.000	0.697	0.021	0.335	Partial
TS	1.000	0.685	0.140	0.255	Full

Panel B. Multigroup Analysis of Structural Path Coefficients.						
Relationship	Saudi β	Non-Saudi β	*Δ*β	Bootstrap MGA p	Permutation p	Conclusion
EE → OCB	0.266	0.231	0.035	0.374	0.381	No significant difference
EE → OT	0.094	0.310	−0.216	0.056	0.050	Borderline
EE → TS	0.181	0.605	−0.424	0.001	0.002	Significant
OT → OCB	0.374	0.287	0.087	0.269	0.289	No significant difference
TS → OCB	0.216	0.402	−0.186	0.139	0.146	No significant difference
TS → OT	0.624	0.445	0.178	0.072	0.057	No significant difference

As shown in Table 8—Panel B, Bootstrap and permutation MGA yielded largely consistent results. No significant nationality-based differences emerged for the core trust-related paths. Although TS→OT was numerically stronger among Saudi employees (*β* = 0.624) than among non-Saudi employees (*β* = 0.445), the difference was nonsignificant (*Δβ* = 0.178; bootstrap *p* = 0.072; permutation *p* = 0.057). Likewise, no significant differences were found for TS→OCB (*Δβ* = −.186; *p* = 0.139/.146) or OT→OCB (*Δβ* = 0.087; *p* = 0.269/.289), indicating that the core trust-transfer relationships were broadly comparable across nationality groups.

For EE-related paths, EE→OCB showed no significant group difference (*Δβ* = 0.035; *p* = 0.374/.381), whereas EE→OT provided only borderline evidence of heterogeneity (*Δβ* = −.216; *p* = 0.056/.050). In contrast, EE→TS differed significantly between groups (*Δβ* = −.424; *p* = 0.001/.002), with a stronger relationship among non-Saudi employees (*β* = 0.605) than among Saudi employees (*β* = 0.181). However, because EE did not establish compositional invariance, this difference is treated as exploratory rather than definitive evidence of nationality-based heterogeneity. Overall, the results provide no robust evidence that the central trust-transfer mechanism differs across Saudi and non-Saudi employees.

## 7 Discussion

The present study aims to provide a comprehensive explanation of the relationship between employee engagement (EE) and organizational citizenship behavior (OCB) by examining the mediating roles of trust in the supervisor (TS) and organizational trust (OT). The findings indicate that both parallel and sequential mediation are present. In parallel mediation, engagement influences OCB through both trust dimensions simultaneously, whereas in sequential mediation, engagement influences OCB through interpersonal trust and organizational trust in sequence (47, 68, 75). This study builds on prior research by conceptualizing engagement as a multilevel relational process rather than an individual psychological state, and by incorporating Social Exchange Theory (SET), Leader–Member Exchange (LMX) theory, and Trust Transfer Theory.

The direct effect of EE on OCB highlights that high levels of EE can motivate employees to go above and beyond what is expected in their jobs to contribute to organizational effectiveness (9, 101). This aligns with engagement theory developed by Kahn (48) and with research by Saks (50), Jung et al. (102), Hasibuan and Farisi (9), and Cristian and Nuvriasari (3), which shows that the dimensions of vigor, dedication, and absorption lead to intrinsic motivation for discretionary actions. From the SET perspective, these behaviors stem from reciprocity; employees respond to supportive and meaningful work contexts with extra-role behaviors (35). In the healthcare industry, which demands teamwork and collaboration to provide quality care, this is particularly relevant (74).

The results showed that EE had a significant positive effect on TS and OT, affirming that when employees are engaged, their perception of workplace relationships is more positive (29, 34). In accordance with LMX theory, high-quality supervisor–employee relationships foster trust, respect, and support between supervisors and employees, strengthening interpersonal relationships (103). Notably, EE had a greater impact on TS than on OT, indicating that trust develops at the individual-interpersonal level before moving to the organizational level. This trend aligns with the notion that when individuals form overall organizational perceptions, they first consider their direct relational context (57).

The results support the Trust Transfer Theory, as the correlation between trust in supervisors (TS) and organizational trust (OT) is strong. Supervisors represent the organization, and employees are likely to mirror their relationship with supervisors in their perceptions of the organization (17, 42). This process is especially prominent in the healthcare sector, where supervisors manage activities, translate policies, and link management with staff activities (103). Supervisors are particularly important in Saudi Arabia's changing private healthcare system under Vision 2030, as they significantly shape employees’ perceptions of fairness, dependability, and organizational support (19).

The complementary relationships between interpersonal and institutional trust (29) are evident in the significant prediction of OCB by both TS and OT. Trust in supervisors is a stronger predictor of interpersonal citizenship behaviors (helping behaviors, knowledge sharing, and team cooperation), whereas trust in the organization is a stronger predictor of organizational citizenship behaviors (organizational loyalty, compliance, and advocacy) (34, 63). In contexts like health care, where resources are scarce and competition is high, institutional trust is especially important for sustaining long-term voluntary contributions (57).

The results indicate that employee engagement alone is insufficient to sustain organizational citizenship behavior (OCB); its impact on OCB must be linked to trust at both the individual and organizational levels. The mediating roles of trust in the supervisor (TS) and organizational trust (OT) underscore trust's function as a psychological mechanism linking engagement to discretionary performance (30, 69). In a psychologically safe, trusting environment, employees are more likely to go the extra mile, but this is only possible when they are engaged (56). Even among high-engagement employees, a restricted range of action can be expected in low-trust environments, as previous studies have shown (57). This is especially true in the medical field, where trust is paramount to offset uncertainty, reliance, and performance demands. Interpersonally, TS fosters psychological safety, enabling collaboration, knowledge sharing, and proactive action, particularly in hierarchical environments where supervisors play a significant role (30). In addition, it encourages interpersonal risk-taking, which is essential for collaborative work and for ensuring patient safety (101). On the organizational level, OT reflects general fairness and benevolence, facilitating the continuation of OCB norms.

The serial mediation pathway (EE → TS → OT → OCB) provides strong evidence for a cascading trust model (41). Engagement helps build trust in a supervisor, and consequently in the organization, leading to increased OCB (104, 105). This aligns with Trust Transfer Theory and SET, which show how micro-level relationship experiences influence macro-level organizational experiences and behaviors (52). This suggests a potential multiplicative effect of investing in leadership skills and relationship-building, as it not only enhances TS but also OT and OCB (54).

Although these indirect pathways are very strong, the direct effect of EE on OCB remains significant and persistent, suggesting that indirect effects are only partial. This suggests an additional connection between engagement and discretionary behaviors, though not the sole one, and that trust plays a role in that connection. Other factors, such as psychological empowerment (106), organizational identification (29), perceived organizational support (101), psychological safety (56), and job satisfaction (107), are likely to be contributing factors. Future studies should include these variables in the model and explore boundary conditions, such as cultural values, employment status, and organizational characteristics (108).

Beyond the overall structural and mediation effects, the nationality-based analysis offers additional evidence on the generalizability of the proposed trust-transfer mechanism. Bootstrap and permutation MGA revealed no significant differences between Saudi and non-Saudi employees in the TS→OT, TS→OCB, or OT→OCB relationships, suggesting that the core process by which trust in supervisors extends to organizational trust and promotes citizenship behavior is broadly consistent across nationality groups. Although EE→TS was significantly stronger among non-Saudi employees, this finding should be considered exploratory because EE did not establish compositional invariance. Likewise, the borderline difference in EE→OT provides insufficient evidence of systematic nationality-based heterogeneity. These findings are particularly relevant to Saudi Arabia's private healthcare sector, which is characterized by rapid growth, professionalized workforces, and substantial expatriate participation (4). While employment arrangements, external career opportunities, and relational expectations may shape how employees develop workplace trust (109), the present findings indicate that the central trust-transfer process remains relatively stable across nationality groups. In an environment marked by staff turnover and competing demands for profitability and patient care (110), consistent supervisory communication, fairness, and supportive behavior may therefore provide broadly applicable foundations for building organizational trust and encouraging citizenship behavior (41).

Overall, this study advances theory by proposing a multilevel trust relationship that links employee engagement to OCB through SET and LMX theory. The model explains 54.5% of the variance in OCB, a substantial amount. It also shows that when employees are engaged, they can initiate a chain of engagement by building trust with their supervisors, which in turn fosters trust in the organization and ultimately results in discretionary behavior. Overall, these findings provide a valuable perspective on fostering high-trust, high-performance organizations within Saudi Arabia's healthcare system, especially in the context of the country's growing private healthcare sector, which seeks to improve patient outcomes and employee satisfaction (68).

## 8 Implications

The study provides valuable theoretical implications for the organizational behavior literature. It advances theory by integrating Social Exchange Theory (SET), Leader-Member Exchange (LMX) Theory, and Trust Transfer Theory into a single model that explains how employee engagement leads to organizational citizenship behavior (OCB) through multiple levels of trust. The significant serial mediation pathway indicates that trust in the supervisor generalizes to the organization, making supervisors representatives of the organization and influencing overall organizational trust perceptions (37, 41), thereby supporting Trust Transfer Theory. Therefore, trust is a “cascading relational resource” rather than merely an individual psychological attribute. Furthermore, the results contribute to the employee engagement literature by showing that engagement with supervisors and organizational trust serve as complementary mediators in the relationship with OCB. Both direct and indirect effects are significant, indicating partial mediation and that engagement indirectly influences citizenship behavior through trust-based mechanisms and other motivational factors. These findings contradict theories based on a single mediator and align with evidence that psychological capital and intrinsic motivation also contribute to OCB (104). Accordingly, this study contributes to the multilevel trust literature by conceptualizing trust as a multilevel process. Building trust in supervisors will increase organizational trust, which will consequently improve organizational legitimacy, psychological safety, and citizenship behavior (29, 40), providing a solid theoretical foundation for future multilevel trust studies.

The results have important implications and suggestions for private healthcare HR managers and HRM in Saudi Arabia under Vision 2030. First, they underscore the strategic importance of supervisors who build high-quality, trusting relationships with employees. Healthcare organizations must cultivate leadership skills, integrity, and transparent communication to foster trust between leadership and employees, leading to OCB (34, 68). Second, organizations should enhance justice and implement transparent mechanisms for performance appraisal, promotion, and rewards. Employees’ perceptions of distributive, procedural, and interactional justice lead to trust in their supervisors and the organization, prompting greater satisfaction, engagement, and citizenship behaviors (110). Given the diverse nature of the private healthcare workforce in Saudi Arabia, organizations should also use targeted trust-building strategies that acknowledge differences in backgrounds, career goals, and employment conditions, and ensure equitable treatment and clear organizational decision-making (111). Third, healthcare organizations can create a psychologically safe work environment where employees can voice concerns without fear of negative repercussions and share ideas. To establish conducive, safe environments for sharing, knowledge exchange, and proactive patient safety measures, supervisors must be trained to develop these skills (56). This should increase employees’ engagement, trust, and discretion, improving organizational resilience and ultimately influencing patient care and health outcomes (9).

## 9 Limitations and future research directions

This study clarifies the multilevel trust mechanisms linking employee engagement to OCB and identifies key limitations. First, this cross-sectional study cannot draw causal conclusions or establish the order of trust-transfer processes (24). Future studies might consider using longitudinal designs to strengthen causal inferences and gain a deeper understanding of how trust is built across organizational levels. Second, the results may be limited by the convenience sample and by confining the study to private health care organizations in Riyadh and the Eastern Province. Future studies could examine the mediation mechanism across sectors, regions, and cultures, especially in non-Western contexts, where trust dynamics may differ and hierarchical interrelationships may be stronger (39, 112–114). Third, other psychological constructs besides trust may mediate the engagement-OCB relationship, such as psychological empowerment, organizational identification, and perceived organizational support (106). Moreover, leadership styles vary across healthcare environments and supervisory groups (112), indicating the need for leadership development in specific settings. Finally, distinguishing between expatriate and national employees may provide insights into contractual expectations and cultural differences in the process of trust development, which is relevant for MNOs implementing Vision 2030 reforms (115).

## Data Availability

The datasets generated and analyzed during the current study are available from the corresponding author upon reasonable request.

## References

[1] Humayun KF Naveed J. The factors affecting productivity in healthcare sector. Int J Nurs Care. (2024) 12(2):34–43. 10.37506/6dsvps24

[2] Schaufeli WB Salanova M González-Romá V Bakker AB. The measurement of engagement and burnout: a two-sample confirmatory factor analytic approach. J Happiness Stud. (2002) 3(1):71–92. 10.1023/A:1015630930326

[3] Cristian MBP Nuvriasari A. The effect of employee engagement, organizational commitment, and work motivation on organizational citizenship behavior. Arthatama J Bus Manag Account(2025) 9(2):480–93. 10.54518/art.9.2.2025.966

[4] Al Ghamdi K Alammari D (2025). Effect of leadership styles on employee experience among healthcare professionals: a systematic review with implications for Saudi Arabia's Vision 2030. Int J Innov Res Sci Stud, 8(12), 251–68. 10.53894/ijirss.v8i12.11067

[5] Geese F Schmitt KU (2023). Interprofessional collaboration in complex patient care transition: a qualitative multi-perspective analysis. Healthcare. 2023; 11 (3):359. 10.3390/healthcare11030359 36766934 PMC9914692

[6] Bardhan S Haque S. Empowering healthcare: the link of self-efficacy, employee engagement, and citizenship behaviors. Dev Learn Organ Int J. (2025) 39(6):33–6. 10.1108/DLO-09-2024-0249

[7] Organ DW. Organizational citizenship behavior: the good soldier syndrome. Lexington Books/DC Heath and com. (1988).

[8] Podsakoff PM MacKenzie SB Paine JB Bachrach DG. Organizational citizenship behaviors: a critical review of the theoretical and empirical literature and suggestions for future research. J Manage. (2000) 26(3):513–63. 10.1177/014920630002600307

[9] Hasibuan JS Farisi S. Determinants of employee engagement and organizational citizenship behavior of nurses in public hospitals in Indonesia. Probl Perspect Manag. (2025) 23(1):584. 10.21511/ppm.23(1).2025.44

[10] Khajoei R Jokar M Vasli P. Nurses’ experiences of the attributes of organizational citizenship behavior: a qualitative content analysis. BMC Health Serv Res. (2024) 24(1):538. 10.1186/s12913-024-10939-8 38671514 PMC11055363

[11] Zeng W Ma S Wu L Wang C Huang Y Li B. Group citizenship behavior in healthcare organization, doctor-patient relationship, work engagement and turnover intention: a moderated mediation model. Int J Health Plann Manage. (2023) 38(3):687–701. 10.1002/hpm.3615 36805680

[12] Ansong A Addison RA Yeboah MA Ansong LO. Relational leadership and organizational citizenship behavior: do employee well-being and employee voice matter? Leadersh Health Serv. (2024) 37(2):259–76. 10.1108/LHS-06-2023-0041 37934205

[13] Niwes A Kulachai W Wannarak J Homyamyen P. Unleashing potential: the impact of supervisor trust on organizational citizenship and job performance. Edelweiss Appl Sci Technol. (2024) 8(6):1517–28. 10.55214/25768484.v8i6.2269

[14] Xu Q Liu S Huang H. Psychological empowerment and challenge-oriented organizational citizenship behavior: a dual process model. Front Psychol. (2024) 15:1432260. 10.3389/fpsyg.2024.1432260 39726614 PMC11670482

[15] Kopuz K Ozisli O Ekmen E Kocak O (2026). The role of trust and happiness in the relationship between organizational ostracism and turnover intentions among healthcare professionals: a serial mediation model. BMC Health Serv Res*,* 26:217. 10.1186/s12913-026-14039-7 41545996 PMC12892542

[16] UI Hassan FS Ikramullah M. Transformational leadership and employees’ work engagement: the simple and parallel mediation of self-efficacy and trust in the leader. J Organ Effect People Perform. (2024) 11(2):448–65. 10.1108/JOEPP-09-2022-0275

[17] Aldabbas H Elamin AM Ahmed AZ Gernal L. Building positive workplace behaviors: how trust in the manager enhances sportsmanship behaviors through organizational trust. Front Psychol. (2026) 17:1828581. 10.3389/fpsyg.2026.1828581 42516195 PMC13402171

[18] Kidron A Vinarski Peretz H. The influence of organizational trust and social exchange on organizational citizenship behavior: the mediating role of workplace spirituality. J Organ Effect People Perform. (2026) 13(1):129–46. 10.1108/JOEPP-08-2024-0410

[19] Al-Anezi FM. Challenges of healthcare systems in Saudi arabia to delivering vision 2030: an empirical study from healthcare workers perspectives. J Healthc Leadersh. (2025) 17:173–87. 10.2147/JHL.S516159 40357423 PMC12067643

[20] Mani ZA Goniewicz K. Transforming healthcare in Saudi arabia: a comprehensive evaluation of vision 2030's Impact. Sustainability. (2024) 16(8):3277. 10.3390/su16083277

[21] Singh D Tiwari A Mahajan R Kumar N. Healing the healers: how empathy, active coping and mindfulness drive well-being and happiness of healthcare employees. Int J Sociol Soc Policy. (2026) 46(1-2):1–22. 10.1108/IJSSP-04-2025-0260

[22] Na-Nan K Kanthong S Joungtrakul J. An empirical study on the model of self-efficacy and organizational citizenship behavior transmitted through employee engagement, organizational commitment, and job satisfaction in the Thai automobile parts manufacturing industry. J. Open Innov.: Technol., Mark. Complex. (2021) 7(3):170. 10.3390/joitmc7030170

[23] Khalifa G. Intervening role of supervisor trust and leader-member exchange: an investigation into the role of supervisor support on employee innovative behavior. J Assoc Arab Univ Tour Hosp. (2019) 17(3):46–67. 10.21608/jaauth.2020.40843.1070

[24] Lampogo J Andoh N Osei DD Mukhtar MI Brenya R Zoogah BR (2026). Ethical leadership and employee proactivity: the serial mediation of trust, psychological safety and psychological empowerment (no. Hal-05555317). J Econ Manag Trade, 32(3), 59–71. 10.9734/jemt/2026/v32i31403

[25] Amjad S Yasin R Munir Y Atif M. Knowledge-oriented leadership, organizational trust and psychological capital: a gateway to innovative performance: a multisource approach. Manag Res Rev. (2025) 48(8):1231–56. 10.1108/MRR-06-2024-0468

[26] Kumar R Singh A Kassar ASA Humaida MI Joshi S Sharma M. Adoption challenges to artificial intelligence literacy in public healthcare: an evidence-based study in Saudi arabia. Front Public Health. (2025) 13:1558772. 10.3389/fpubh.2025.1558772 40371275 PMC12076014

[27] Rajâa O Mekkaoui A. Revealing the impact of social exchange theory on financial performance: a systematic review of the mediating role of human resource performance. Cogent Bus Manag. (2025) 12:2475983. 10.1080/23311975.2025.2475983

[28] Thiel CE Hardy III Peterson JH Welsh DR Bonner DT M J. Too many sheep in the flock? Span of control attenuates the influence of ethical leadership. J Appl Psychol. (2018) 103(12):1324–34. 10.1037/apl0000338 30024198

[29] Dai Y Tang YM Chen W Hou J. How organizational trust impacts organizational citizenship behavior: organizational identification and employee loyalty as mediators. Front Psychol. (2022) 13:996962. 10.3389/fpsyg.2022.996962 36457918 PMC9706095

[30] Numniyom P Nawakitphaitoon K Chungsawanant P. Diversity management and organizational citizenship behavior in Thai public utilities: examining the dual mediating effects of organizational justice and organizational trust. Empl Relat. (2026) 48(4):630–50. 10.1108/ER-10-2025-0904

[31] AlDayel WA Alhaider M Abid K Alsarhan F. Institutionalism and talent management challenges in Saudi Arabia's Public healthcare sector. Empl Relat. (2025) 47(6):907–27. 10.1108/ER-11-2024-0677

[32] Suleiman AK Ming LC. Transforming healthcare: saudi Arabia's Vision 2030 healthcare model. J Pharm Policy Pract. (2025) 18(1):2449051. 10.1080/20523211.2024.2449051 39845746 PMC11753010

[33] Blau PM. Exchange and Power in Social Life. New York: John Wiley (1964).

[34] Raziq MM Wazir R Memon MA Rice JL Moazzam M. Empowering leadership, employee organizational commitment, and organizational citizenship behavior: the roles of leader authenticity and trust. Int J Prod Perform Manag. (2025) 74(1):81–106. 10.1108/ijppm-09-2023-0489

[35] Mallett X. Investigating the application of social exchange theory in enhancing workplace relationships and employee job satisfaction. Int Acad J Organ Behav Hum Resour Manag. (2025) 12(3):35–42. 10.71086/IAJOBHRM/V12I3/IAJOBHRM1212

[36] Colquitt JA Scott BA Rodell JB Long DM Zapata CP Conlon DE. Justice at the millennium, a decade later: a meta-analytic test of social exchange and affect-based perspectives. J Appl Psychol. (2013) 98(2):199–236. 10.1037/a0031757 23458336

[37] Kim J Choi W Yoon J. How transformational and ethical leadership promote organizational citizenship behavior: the roles of change-related self-efficacy and leader-member exchange. Leadersh Organ Dev J. (2025) 46(3):449–64. 10.1108/lodj-09-2024-0570

[38] Gerstner CR Day DV. Meta-analytic review of leader–member exchange theory: correlates and construct issues. J Appl Psychol. (1997) 82(6):827–44. 10.1037/0021-9010.82.6.827

[39] Dawuda A Amadu S Adam AR Akabutu C Abdallah MH Fianku B. Impact of leader-member exchange (LMX) on organizational performance: synthesis of literature (2015-25). Hum Resour Leadersh J. (2026) 11(1):39–57. 10.47941/hrlj.3499

[40] Teklehaimanot ML Bekele E Mekonnen TW. Be a people-centered leader and foster organizational citizenship behavior: the sequential mediation of leader-member exchange and employee satisfaction in a transitioning economy. Int J Public Leadersh (2026) 22(2):188–204. 10.1108/ijpl-03-2025-0045

[41] Basit AA. Trust in supervisor and job engagement: mediating effects of psychological safety and felt obligation. J Psychol. (2017) 151(8):701–21. 10.1080/00223980.2017.1372350 29043913

[42] Lee MCC Lin MH Srinivasan PM Carr SC. Transformational leadership and organizational citizenship behavior: new mediating roles for trustworthiness and trust in team leaders. Curr Psychol. (2024) 43(11):9567–82. 10.1007/s12144-023-05095-x

[43] Kartikasari ED Wahyuni P. Influence of leader-member exchange on organizational citizenship behavior mediated by employee engagement at the regional development planning agency yogyakarta. Strata Business Review. (2025) 3(1):32–45. 10.59631/sbr.v3i1.347

[44] Hassan MA Ying Z Shahrulnizam NAA Wan Mohd Yunus WMA Abdul Kohar UH Nor DAM. A systematic literature review on the impact of leader-member exchange (LMX) and nurse morality in the healthcare sector. Int J Res Innov Soc Sci. (2025) 9(3):1936–52. 10.47772/ijriss.2025.90300152

[45] Pragha P Natarajan T Dhalmahapatra K. Adoption of NFT transaction in metaverse platform: a trust transfer approach to leverage trust among users. Digit Policy Regul Gov. (2025) 27(4):422–45. 10.1108/DPRG-08-2024-0178

[46] Suleman D Wianti W Sofyanty D Haryati RA Joesah N Hakim L. Trust transfer in social commerce: the role of platform trust and seller trust in shaping consumer behavior in Indonesia. Dinasti Int J Educ Manag Soc Sci. (2026) 7(4):3882–93. 10.38035/dijemss.v7i4.6598

[47] Zhang X Gao Y. Organizational culture, trust, and supervisor support: drivers of employee commitment in China. Front Psychol. (2025) 16:1548514. 10.3389/fpsyg.2025.1548514 41394056 PMC12695608

[48] Kahn WA. Psychological conditions of personal engagement and disengagement at work. Acad Manage J. (1990) 33(4):692–724. 10.2307/256287

[49] Sonnentag S. A task-level perspective on work engagement: a new approach that helps to differentiate the concepts of engagement and burnout. Burn Res. (2017) 5:12–20. 10.1016/j.burn.2017.04.001

[50] Saks AM. Antecedents and consequences of employee engagement revisited. J Organ Effect People Perform. (2019) 6(1):19–38. 10.1108/JOEPP-06-2018-0034

[51] Razkiandra R Rositawati S. Pengaruh perceived organizational support terhadap work engagement pada barista starbucks di kota bandung. Bandung Conf Ser Psychol Sci. (2024) 4(1):628–34. 10.29313/bcsps.v4i1.10322

[52] Chen JK Sriphon T. Authentic leadership, trust, and social exchange relationships under the influence of leader behavior. Sustainability. (2022) 14(10):5883. 10.3390/su14105883

[53] Robinson SL. Trust and breach of the psychological contract. Adm Sci Q. (1996) 41(4):574–99. 10.2307/2393868

[54] Akter KM Banik S Tang SM Adnan Z. Transformational leadership and climate of trust: mediating role of organizational justice. SAGE Open. (2024) 14(4):21582440241293132. 10.1177/21582440241293132

[55] Braun BI Bozikis MR Casey ML Weissman DN Orze KJ Riehle A. Improving healthcare workforce and patient safety: a scoping meta-review. Workplace Health Saf. (2026) 74(10):534–57. 10.1177/21650799261446613 42244411

[56] Zuhaena F Cahyo H Arinastuti. Employee engagement and organizational citizenship behavior in SMEs: the mediating role of psychological safety. Int J Econ Bus Account Res (IJEBAR). (2025) 9(4). 10.29040/ijebar.v9i4.19982

[57] Mach M Abrantes ACM Alvarado JR. The role of perceived human resource management practices in organizational citizenship behaviors: evidence from the healthcare sector. Empl Relat Int J. (2025) 47(1):127–47. 10.1108/er-07-2023-0386

[58] Lee H Jung T. The impact of coaching leadership and negative leadership on organizational attachment and job performance: through mediating effects of intrinsic motivation, trust in supervisor, and job autonomy. Korean Ment Health Behav Anal Assoc. (2024) 7(2):83–111. 10.70316/kmba.2023.7.2.83

[59] Tahir U Hammad A Majeed MI. Transformational leadership and healthcare project success: the mediating role of organizational trust in healthcare projects in Pakistan. Phys Educ Health Soc Sci. (2026) 4(1):1–16. 10.63163/jpehss.v4i1.1309

[60] Jatoi SA Channar SH. When does trust in leader foster innovative work behavior in public hospitals? Role of employee engagement and high-performance work systems. J Health Organ Manag. (2026):1–21. 10.1108/JHOM-08-2025-0498 41992577

[61] Nohe C Hertel G. Transformational leadership and organizational citizenship behavior: a meta-analytic test of underlying mechanisms. Front Psychol. (2017) 8:1364. 10.3389/fpsyg.2017.01364 28848478 PMC5554340

[62] Espinoza L Leal SR Herrera CV Pérez F Acosta H. A meta-analytic examination of the association between vertical and horizontal trust and the in-role, extra-role, and organizational levels of performance. Psychol Soc Educ. (2025) 17(2):26–43. 10.21071/pse.v17i2.17570

[63] Yesildag AY Ugurluoglu Aldogan E. Assessment of the relationships between leader behavior, organizational silence, work engagement, and extra role behaviors in hospitals. BMC Health Serv Res. (2026) 26:881. 10.1186/s12913-026-14652-6 42092920 PMC13317359

[64] Huang N Qiu S Yang S Deng R. Ethical leadership and organizational citizenship behavior: mediation of trust and psychological well-being. Psychol Res Behav Manag. (2021) 14:655–64. 10.2147/PRBM.S311856 34104011 PMC8179798

[65] Liao EY Chun H. Supervisor monitoring and subordinate innovation. J Organ Behav. (2016) 37(2):168–92. 10.1002/job.2035

[66] Akram A Kamran M Iqbal MS Habibah U Atif Ishaq M. The impact of supervisory justice and perceived supervisor support on organizational citizenship behavior and commitment to supervisor: the mediating role of trust. Cogent Bus Manag. (2018) 5:1493902. 10.1080/23311975.2018.1493902

[67] Iddrisu I. Understanding the nexus between organizational culture and trust: the mediating roles of communication, leadership, and employee relationships. Sustain Futures. (2025) 9:100555. 10.1016/j.sftr.2025.100555

[68] Almarhabi MA Alrawi MA. The impact of adopting transformational leadership practices on employees’ commitment in Saudi Arabia's Health sector: 2024–2025. J Econ Adm Leg Sci. (2026) 10(3):95–105. 10.26389/AJSRP.H161025

[69] Lorenz T. Ethical leadership: a multi-stage mediation model of value congruence and organizational identification on employee engagement. Adm Sci. (2025) 15(9):329. 10.3390/admsci15090329

[70] Manzoor SR Ullah A Ullah R Khattak A Han H Yoo S. Micro CSR intervention towards employee behavioral and attitudinal outcomes: a parallel mediation model. Humanit Soc Sci Commun. (2023) 10(1):1–14. 10.1057/s41599-023-02433-z

[71] Akgerman A Gül D Sönmez B. The relationship between inclusive leadership, organizational justice, work engagement, and organizational citizenship behavior in healthcare workers. Leadersh Health Serv. (2025) 38(2):192–210. 10.1108/LHS-05-2024-0042 39656201

[72] Worku MA Debela KL. A systematic literature review on organizational citizenship behavior: conceptualization, antecedents, and future research directions. Cogent Bus Manag. (2024) 11:2350804. 10.1080/23311975.2024.2350804

[73] Nguyen DT Teo ST Halvorsen B Staples W. Leader humility and knowledge sharing intention: a serial mediation model. Front Psychol. (2020) 11:560704. 10.3389/fpsyg.2020.560704 33424676 PMC7794012

[74] Tarroza RAT Organia EG. Mediating effect of employee engagement on the relationship between triple bottom line application and organizational citizenship behavior. Int J Multidiscip Res. (2026) 8(3). 10.36948/ijfmr.2026.v08i03.76370

[75] Chen SY Ahlstrom D Uen JF. Organizational trust and employee work outcomes: a moderated mediation model. Curr Psychol. (2025) 44(8):6565–78. 10.1007/s12144-025-07626-0

[76] Yamaura K Okada S Nishihara Y Shiozawa N Mukai E Sakaue Y. Revisiting leader–member exchange: a review through the lens of the “too much of a good thing” effect. Front Psychol. (2026) 17:1722767. 10.3389/fpsyg.2026.1722767 41947964 PMC13050747

[77] Al-Faouri EH Abu Huson Y Aljawarneh NM Alqmool TJ. Smart human resource management and innovation performance. Sustainability. (2024) 16:4747. 10.3390/su16114747

[78] Elamin AM. Exploring the relationship between perceived organizational support and affective commitment among Saudi employees: the mediating effect of organizational trust. Bus Theory Pract. (2024) 25(2):434–46. 10.3846/btp.2024.16845

[79] Obilor EI. Convenience and purposive sampling techniques: are they the same. Int J Innov Soc Sci Educ Res. (2023) 11(1):1–7.

[80] Kang H. Sample size determination and power analysis using the G*power software. J Educ Eval Health Prof. (2021) 18:17. 10.3352/jeehp.2021.18.17 34325496 PMC8441096

[81] Stefana A Damiani S Granziol U Provenzani U Solmi M Youngstrom EA. Psychological, psychiatric, and behavioral sciences measurement scales: best practice guidelines for their development and validation. Front Psychol. (2025) 15:1494261. 10.3389/fpsyg.2024.1494261 39916786 PMC11798685

[82] Schaufeli WB Bakker AB Salanova M. The measurement of work engagement with a short questionnaire: a cross-national study. Educ Psychol Meas. (2006) 66(4):701–16. 10.1177/0013164405282471

[83] Alzuman A Jaafari MA Ali Z Ali R Bakadam LI. Development and validation of the organizational health behavior index: a mixed-methods instrument for measuring organizational health. Front Psychol. (2025) 16:1689559. 10.3389/fpsyg.2025.1689559 41409059 PMC12705562

[84] Niehoff BP Moorman RH. Justice as a mediator of the relationship between methods of monitoring and organizational citizenship behavior. Acad Manage J. (1993) 36(3):527–56. 10.5465/256591

[85] Tziner A Sharoni G. Organizational citizenship behavior, organizational justice, job stress, and work-family conflict: examination of their interrelationships with respondents from a non-western culture. Rev Psicol Trab Organ. (2014) 30(1):35–42. 10.5093/tr2014a5

[86] Elamin AM Tlaiss HA. Exploring the relationship between organizational citizenship behavior and organizational justice in the islamic Saudi Arabian context. Empl Relat Int J. (2015) 37(1):2–29. 10.1108/ER-03-2014-0033

[87] Kabasakal H Dastmalchian A Imer P. Organizational citizenship behavior: a study of young executives in Canada, Iran, and Turkey. Int. J. Hum. Resour. Manag. (2011) 22(13):2703–29. 10.1080/09585192.2011.599943

[88] LePine JA Erez A Johnson DE. The nature and dimensionality of organizational citizenship behavior: a critical review and meta-analysis. J Appl Psychol. (2002) 87(1):52–65. 10.1037/0021-9010.87.1.52 11916216

[89] Dalal RS (2005). A meta-analysis of the relationship between organizational citizenship behavior and counterproductive work behavior. J Appl Psychol*,* 90(6), 1241–55. 10.1037/0021-9010.90.6.1241 16316277

[90] Gupta V Singh S. An empirical study of the dimensionality of organizational justice and its relationship with organizational citizenship behavior in the Indian context. Int. J. Hum. Resour. Manag. (2013) 24(6):1277–99. 10.1080/09585192.2012.709188

[91] Podsakoff PM MacKenzie SB Moorman RH Fetter R. Transformational leader behaviors and their effects on followers’ trust in leader, satisfaction, and organizational citizenship behaviors. Leadersh Q. (1990) 1(2):107–42. 10.1016/1048-9843(90)90009-7

[92] Chénard-Poirier LA Vandenberghe C Morin AJ. From perceived supervisor social power to employee commitment: definition and scale development. Front Psychol. (2021) 12:603739. 10.3389/fpsyg.2021.603739 PMC7952328 33716869

[93] Wildman JL Thayer AL Warren C Fiore SM Salas E. Interpersonal trust and distrust at work: scale validation and theoretical exploration. Appl Psychol. (2025) 74(1):e12588. 10.1111/apps.12588

[94] Harris J Strefford P. English medium instruction learners’ self-efficacy, engagement, and satisfaction: developing a measurement instrument. Asia Pac J Educ. (2026) 46(1):80–97. 10.1080/02188791.2024.2341991

[95] Hair JF Risher JJ Sarstedt M Ringle CM. When to use and how to report the results of PLS-SEM. Eur Bus Rev. (2019) 31(1):2–24. 10.1108/EBR-11-2018-0203

[96] Chin W Cheah J Liu Y Ting H Lim X Cham TH. Demystifying the role of causal-predictive modeling using partial least squares structural equation modeling in information systems research. Ind Manag Data Syst. (2020) 120(12):2161–209. 10.1108/IMDS-10-2019-0529

[97] Podsakoff PM MacKenzie SB Lee JY Podsakoff NP. Common method bias. J Appl Psychol. (2003) 88(5):879–903. 10.1037/0021-9010.88.5.879 14516251

[98] Huy PQ Phuc VK. Insight into technologically vigilant leadership: smart sustainable circular supply chain management in the economic context of industry 6.0. Corp Gov Sustain Rev. (2025) 9(4):75–88. 10.22495/cgsrv9i4p7

[99] Ghosh A Mahato S Sen S. Validating writing skill dimensions through structural equation modeling in secondary education. J Glob Res Educ Soc Sci. (2025) 19(3):336–47. 10.56557/jogress/2025/v19i39590

[100] Silva AEO Salazar G. Assessment of scientific competencies in secondary school students: exploring the relationships among inquiry, explanations of the physical world, and the design of technological solutions. Int J Eval Res Educ. (2026) 15(2):1114–28. 10.11591/ijere.v15i2.37351

[101] Sulaimiah AEO Serif S Widiana H Wahyu Z. Employee engagement mediates self-efficacy and perceived organizational support on nurses’ organizational citizenship behavior. J Ilm Manaj Kesatuan. (2025) 13(6):4961–74. 10.37641/jimkes.v13i6.3991

[102] Jung HS Seo KH Yoon HH. The importance of leader integrity on family restaurant employees’ engagement and organizational citizenship behaviors: exploring sustainability of employees’ generational differences. Sustainability. (2020) 12(6):2504. 10.3390/su12062504

[103] Nurjanah N Dasmo D Mailizar M Wati S. Path analysis of organizational climate, servant leadership, and personality on teachers’ OCB: the mediating role of trust. Tarbawi J Keilm Manaj Pendid. (2026) 12(01):1–10. 10.32678/tarbawi.v12i01.12138

[104] Kim MJ Park YJ. Building sustainable organizational citizenship behavior in hospitality: structural relationships of rapport, trust, and psychological capital among airline cabin crew. Sustainability. (2025) 17(23):10804. 10.3390/su172310804

[105] Ahmad R Nawaz MR Ishaq MI Khan MM Ashraf HA. Social exchange theory: systematic review and future directions. Front Psychol. (2023) 13:1015921. 10.3389/fpsyg.2022.1015921 36710813 PMC9878386

[106] Kosar R. A the impact of psychological empowerment on organizational citizenship behavior and knowledge sharing behavior: the mediating role of employee engagement and moderating role of leader-member exchange. Jinnah Bus Rev. (2017) 5(2):1–12. 10.53369/tnyv1870

[107] Zhao Y Ompok CC. Job satisfaction, work engagement, and OCB in preschool teachers: a review of mediating mechanisms. J Manag Pract Humanit Soc Sci. (2025) 9(4):232–43. 10.33152/jmphss-9.4.3

[108] Mankgele KP Fatoki O. Environmentally specific empowering leadership and organizational citizenship behavior toward the environment: the roles of green work engagement, psychological ownership, and environmental concern. Asian Manag Bus Rev. (2025) 5(1):143–59. 10.20885/ambr.vol5.iss1.art10

[109] Naseer M Haq AU Shah SMA. Understanding turnover intentions in Pakistan's Healthcare sector: a qualitative exploration of supervisory behavior, stress, and cultural norms. Annu Methodol Arch Res Rev. (2024) 2(5):1–18. 10.63075/ge4wh586

[110] Alotaibi M. The role of trust in supervisors as a mediator between fairness of appraisal evaluation and employee satisfaction: evidence from the public sector in Saudi arabia. J Manag World. (2024) 2024(5):275–85. 10.53935/jomw.v2024i4.1134

[111] Ali I Ali M Leal-Rodríguez AL Albort-Morant G. The role of knowledge spillovers and cultural intelligence in enhancing expatriate employees’ individual and team creativity. J Bus Res. (2019) 101:561–73. 10.1016/J.JBUSRES.2018.11.012

[112] Wu Y Awang SR Zou D Li W Ahmad T. A meta-analysis of authentic leadership research in healthcare sector. Leadersh Organ Dev J. (2026) 47:1364–87. 10.1108/lodj-08-2025-0700

[113] Gonlepa MK Dilawar S Amosun TS. Understanding employee creativity from the perspectives of grit, work engagement, person-organization fit, and feedback. Front Psychol. (2023) 13:1012315. 10.3389/fpsyg.2022.1012315 36778179 PMC9911672

[114] Kulawardena LKRD Kalinga ML Wijesinghe N. Factors affecting intention to adopt e-commerce in the handicraft industry in Sri Lanka. Asian J Educ Soc Stud. (2026) 52(1):222–40. 10.9734/ajess/2026/v52i12771

[115] Vision 2030 Kingdom of Saudi Arabia. (2016). Saudi Vision 2030. Available online at: https://www.vision2030.gov.sa (Accessed September 10, 2026).

